# Effect of adequate daily water intake versus inadequate water intake on the risk of major chronic diseases in healthy adults: a systematic review protocol

**DOI:** 10.1186/s13643-026-03108-x

**Published:** 2026-03-04

**Authors:** Lavra Nanayakkara, Anna Rangan, Gopala Rangan

**Affiliations:** 1 Micheal Stern Laboratory for Polycystic Kidney Disease, Level 5, Centre for Transplant and Renal Research, Westmead Institute for Medical Research, The University of Sydney, PO Box 412, 176 Hawkesbury Road, Westmead, NSW 2145 Australia; 2 University of New England and University of Newcastle, Joint Medical Program, Armidale, NSW Australia; 3 Sydney Nursing School, Faculty of Medicine and Health, Charles Perkins Centre, The University of Sydney, Sydney, NSW Australia; 4 Department of Renal Medicine, Westmead Hospital, Western Sydney Local Health District, Sydney, NSW Australia

**Keywords:** Water intake, Chronic disease, Cardiovascular, Respiratory, Diabetes, Malignancy, Kidney, Risk

## Abstract

**Background:**

Chronic diseases (cardiovascular, cancer, respiratory, diabetes and chronic kidney disease) account for over 80% of global mortality and remain a leading public health challenge. Although water intake is essential to physiological function and homeostasis, its role in the long-term prevention of chronic diseases remains underexplored. Current guidelines are vague, inconsistent, and aimed at preventing acute dehydration, without robust evidence supporting benefits for chronic disease prevention. This study outlines a preliminary systematic review designed to synthesise existing literature on plain water intake and chronic disease risk, with the aim of laying the groundwork for future public health recommendations.

**Methods:**

This review will include English-language randomised controlled trials, cross-sectional studies, case–control studies, and cohort studies assessing the relationship between plain water intake and the incidence of chronic diseases in healthy adults. Only studies measuring oral plain water consumption will be included. Adequate water intake is defined as 2 to 3.7 l daily, based on current international and national recommendations. Eligible studies must report on the risk of developing one or more major chronic diseases and provide quantifiable outcomes (e.g. odds ratios, or relative risks with 95% confidence intervals). A meta-analysis will be conducted where appropriate, and subgroup and sensitivity analyses will explore potential sources of heterogeneity.

**Discussion:**

This systematic review is the first to collate evidence on the collective relationship between plain water intake and multiple chronic disease outcomes. By adopting a binary classification (adequate versus inadequate intake), this preliminary synthesis aims to generate practical and actionable insights that are accessible to the general public. Recognising the fragmented nature of existing research, the findings will serve as a foundation for future studies to refine thresholds and inform clinical and public health guidelines. Limitations such as self-reported data, residual confounding, exclusion of non-English studies, and variability in intake measurement, will be transparently addressed in the interpretations of results.

**Systematic review registration:**

PROSPERO; CRD420251021321. It can be accessed from https://www.crd.york.ac.uk/PROSPERO/view/CRD420251021321.

**Supplementary Information:**

The online version contains supplementary material available at 10.1186/s13643-026-03108-x.

## Background

Chronic diseases represent a significant public health burden worldwide, with profound implications for population health and healthcare systems [1–3]. Chronic diseases, including cardiovascular disease (CVD), cancer, respiratory diseases, diabetes and chronic kidney disease (CKD), account for over 80% of global mortality [4]. The development of chronic diseases is influenced by numerous factors, including genetic predisposition [5, 6], lifestyle [7–9], environmental [6, 8], and socioeconomic determinants [8–10]. Public health initiatives aimed at primary prevention emphasise mitigating modifiable risk factors, particularly lifestyle components [11, 12]. Classically, these include diet [11, 12], physical activity [11, 12], tobacco [12, 13] and alcohol consumption [12, 14, 15] while the role of water intake remains underexplored [16–19].

Water intake is essential for human health, playing a critical role in maintaining homeostasis and supporting physiological functions [20, 21]. Dehydration can cause life-threatening complications within days [22]. Emerging evidence suggests that chronic inadequate hydration may increase risk for specific individual diseases, such as impaired glucose tolerance [18, 23–26]. However, the collective role of water intake in chronic disease prevention has not been systematically investigated. This gap may partly reflect the dynamic nature of individual water requirements, which vary by age [17, 18, 26], sex [17–19, 26, 27], physical activity [18, 27], and climate [18, 27].

The Australian Dietary Guidelines acknowledge that “water is essential to life”, but refrain from quantifying specific recommendations, instead advise consumption of “plenty of water” [16]. International guidelines also provide vague and variable intake ranges [17, 18, 28], developed with consideration to age [17, 18, 28], sex [17, 18, 28], geographical location [17, 18], physical activity [17, 18], climate [17, 18], dietary factors [17, 18] and pathophysiological conditions [17, 18, 28]. These recommendations are primarily based on population medians and focus on preventing acute dehydration; potential benefits of water intake on long-term health outcomes remain uncertain [18].

This systematic review defines adequate water intake as 2 to 3.7 l per day, reflecting the minimum and maximum values of current guidelines [17, 18, 28]. The European Food Safety Authority recommends 2 l per day for women and 2.5 l for men [17]. The Australian National Health and Medical Research Council (NHMRC) and the New Zealand Ministry of Health (MoH) recommend 2.1 l per day for women and 2.6 l for men [28]. The book *Dietary Reference Intakes for Water, Potassium, Sodium, Chloride, and Sulfate* recommends a range of 2.7 to 3.7 l per day for adults [18]. Furthermore, a study published in the *European Journal of Nutrition* reports an adequate intake range of 2.4 to 3.6 l daily for both sexes [19]. Given the close proximity of these recommendations, this review adopts the broadest combined range, 2 to 3.7 l per day, as the operational definition of adequate water intake.

While existing literature often employs a dose–response framework [24, 29, 30], our study deliberately focuses on the binary classification of adequate versus inadequate water intake. This reflects the preliminary and foundational nature of the review, which aims to synthesise a fragmented evidence base to inform future guideline development. Current intake recommendations are often vague and inconsistent; research suggests that such ambiguity in health guidelines can hinder public health effectiveness [31], contribute to poorer outcomes [32], and fail to drive meaningful changes in practice [33]. Our aim is to provide early, evidence informed insights into adequate water intake thresholds that are practical and easily understood by the general public. A binary classification offers simplified, actionable findings that can serve as a foundation for further research. Given this objective, a dose–response or gradient-based approach was considered methodologically inappropriate at this stage, as it risks perpetuating ambiguity in existing guidelines. We acknowledge that further research will be necessary to refine these thresholds and explore more nuanced dose–response relationships. However, this preliminary review seeks to establish clear, methodologically transparent foundation to support such investigations.

The aim of this systematic review is to test the hypothesis that meeting recommended water intake (2 to 3.7 l daily) is inversely associated with risk of developing chronic diseases, namely CVD, cancer, respiratory diseases, diabetes, and CKD, in the healthy adult population. The specific aims are to (i) determine whether meeting these intake targets reduce chronic disease risk, and (ii) perform a meta-analysis to quantitatively assess this relationship.

## Methods

This systematic review protocol has been developed in accordance with the Preferred Reporting Items for Systematic reviews and Meta-Analyses for Protocols (PRISMA-P) 2015 guidelines and checklist [34].

### Search strategy

A comprehensive search of the literature will be conducted across the following electronic databases: PubMed, Medline, Pre-Medline, Cochrane Library, EMBASE, Web of Science, CINAHL, and Scopus. The search will encompass all publications up to March 2025 and will be restricted to studies published in English. To ensure the inclusion of grey literature, additional searches will be conducted through ProQuest Dissertations and Theses, OpenGrey, and relevant government and health agency websites.

Search strategies will incorporate Medical Subject Headings (MeSH), relevant keywords, and free- text words for water intake, CVD, type 2 diabetes, cancer, and CKD. Search terms will be tailored to the indexing systems and thesauri of each database. Boolean operators (‘AND’ and ‘OR’) will be used to combine search concepts appropriately. All MeSH search terms are outlined in more detail in the supplemental documents (refer to Additional File 1)*.*

An updated search will be conducted prior to finalisation of the review to ensure the inclusion of recently published studies. Additionally, the reference lists of included studies will be manually screened to identify any further relevant publications.

All references retrieved from the search will be imported into EndNote X9 (Clarivate Analytics, USA) for reference and duplicate removal. The refined dataset will be transferred to Covidence (Veritas Health Innovation, Melbourne, Australia; available at www.covidence.org) [35], an online platform recommended by the Cochrane Collaboration for systematic review management. Titles and abstracts will be independently screened to determine the relevance of the study according to the eligibility criteria. A record of excluded studies, along with justification for exclusion, will be maintained.

### Inclusion criteria

Studies will be eligible for inclusion in this systematic review if they meet the following criteria: (i) English-language randomised controlled trials (RCTs), cross-sectional studies, case–control studies, prospective, retrospective, comparative, or single-arm cohort studies conducted on healthy adult populations; (ii) focus exclusively on oral plain water intake as the primary exposure variable; (iii) include a comparator of either no intervention or inadequate water intake; (iv) adequate water intake (defined as 2 to 3.7 l daily) [17, 18, 28]; (v) the primary outcome measure for chronic disease development is included: the risk of developing cancer, CVD, respiratory disease, type 2 diabetes, and/or CKD; (vi) the study provides quantitative outcome measures, including relative risks (RR) or odds ratios (OR), with corresponding 95% confidence intervals (CIs) and *p*-values.

### Exclusion criteria

Non-primary studies without categorical data will be excluded from this systematic review. Studies that assessed overall hydration status using fluid intake or inclusion of beverages other than plain water will also be excluded, as well as those that measured the relationship between water quality and the risk of chronic disease(s) as the primary outcome. Additionally, studies using intake thresholds for plain water outside of the range of 2 to 3.7 l per day will be excluded. There will be no restrictions on the geographical location.

### Primary outcome

The primary outcome of this systematic review is the risk of developing a major chronic disease (CVD, cancer, respiratory diseases, diabetes and CKD), among healthy adult populations. The review will compare individuals meeting recommended daily water intake thresholds (2 to 3.7 l daily) to those with inadequate intake.

Where appropriate, a meta-analysis will be conducted to quantitatively synthesise the findings. The results for each chronic disease outcome will be pooled and illustrated using forest plots. Given the variability in effect measures across study designs, ORs will be used as the standard measure of association, along with corresponding 95% CIs and *p*-values. Where required, RR will be converted to OR using Zhang and Yu’s Approximation Formula [36], and Hazard Ratios (HRs) will be converted using the approximation formula HR × 1.25 [37].

As the screening process for eligible studies has not yet been completed, a prespecified hierarchy for selection of effect estimates (e.g. prioritising adjusted over unadjusted estimates) remains under development. Once screening is finalized, this hierarchy will be further refined to ensure that the most methodologically robust and clinically relevant data and are included. Transparency and reproducibility will be prioritised throughout this process. Ultimately, the findings are intended to inform future research directions and support the development of actionable, evidence-based public health guidance.

### Data extraction

Data extraction will be conducted independently by two reviewers (LN, GR) using a standardised data extraction form adapted from the Cochrane Data Collection Form [38]. This form will be tailored to accommodate both randomised and non-randomised studies and to capture all data required for analysis, construction of relevant figures and tables, and quality assessment.

The data collection form will include the following domains: (i) study identification and details (author, year of publication, study design, study ID); (ii) population characteristics (age, sex, smoking status); (iii) main findings and results of the study (effect estimate outcome data including odds ratio, confidence intervals, and *p*-values); (iv) study conclusions; (v) study quality for risk-of-bias assessment.

All water intake volumes will be standardised to litres. Where sufficient data are available, sensitivity analyses will be conducted to evaluate the influence of differing intake assessment methods on the outcomes. Any heterogeneity arising from methodological variations will be evaluated and transparently reported.

All discrepancies will be initially resolved through discussion by the primary authors (LN, GR). If a consensus cannot be reached, a third reviewer (AR) will independently adjudicate in accordance with Chochrane recommendations [38]. Prior to full data extraction, the form will be piloted on a random sample of ten search results, with amendments made as necessary.

For studies with missing or unclear data, corresponding authors will be contacted via email. If data remains unavailable, the study will be included in the qualitative synthesis only and excluded from relevant quantitative meta-analysis. No statistical imputation will be applied. All extracted data will be collated in Microsoft Excel for further analysis and archiving.

### Risk of bias assessment

Observational studies will be assessed using the Newcastle–Ottawa Scale (NOS). The NOS determines the quality of a study based on three domains (i) selection, (ii) comparability, and (iii) assessment of outcome/exposure, employing a star-based system, with a maximum of nine stars awarded overall. In the selection domain (maximum four stars), studies are evaluated on the level of representation of the exposed cohort or case group, the selection of the control group, the ascertainment of exposure or outcome, and in cohort studies, whether the outcome of interest was present at the start of the study. The comparability domain (maximum two stars) assesses the studies ability to control for potential confounding variables. These stars are awarded for studies that use appropriate statistical adjustments or for design elements to mitigate such biases. The assessment of outcome/exposure domain (maximum three stars) considers how the outcome (for cohort studies) or exposure (for case–control studies) was assessed, with emphasis on the reliability and validity of the methods used. In cohort studies, it examines the adequacy of follow-up, while in case–control studies, it assesses the non-response rate. Overall, studies will be evaluated on the number of stars awarded across these domains, where a higher number of stars suggests a lower risk of bias. Randomised control trials will be evaluated using the Cochrane Risk of Bias 2 tool. This tool evaluates bias across several domains, including selection bias, performance bias, detection bias, attrition bias, and reporting bias. Quality assessment will be performed independently by two reviewers (LN, GR) and any differences will be resolved through discussion to reach a consensus. Where necessary, a third reviewer (AR) will be consulted.

Publication bias will be assessed using funnel plot analysis to visually inspect asymmetry in effect estimates. Given the variability in study designs and sample sizes, this method is considered the most appropriate for initial exploration. Where more than ten studies are available for an outcome, Egger’s Regression Test will be conducted to supplement visual interpretation and enhance robustness of our findings. In cases where fewer than 10 studies are available, these tools will be interpreted with caution. The trim-and-fill method will also be applied as a secondary approach to estimate the potential impact of unpublished studies. Selection models will not be used due to their complexity and limited feasibility within the scope of this preliminary review.

### Statistical analysis

OR, 95% CIs and *p*-values will be collectively assessed and reported. Stratified analyses based on potentially confounding factors such as smoking status and geographic region will also be performed if requisite data is available. If sufficient data on water intake volume trends across populations are available, a meta-analysis will be conducted to estimate a population-standard recommendation for adequate water intake, aimed at informing chronic diseases prevention strategies. However, given the expected methodological and clinical heterogeneity across study designs and populations, meta-analysis will only be performed when appropriate. Given the preliminary scope of this review and its focus on clear public health thresholds, dose–response modelling will not be undertaken at this stage. This decision aligns with the binary intake classification adopted to reduce heterogeneity and enhance interpretability. Where meta-analysis is not feasible, narrative synthesis will be employed in accordance with the Synthesis Without Meta-Analysis (SWiM) reporting guidelines [39].

Following careful consideration of methodological differences, subgroup meta-analysis will be conducted to explore potential sources of heterogeneity where possible. The pre-specified subgroup analyses include stratification by study design (randomised control trials vs. observational studies) to account for methodological differences that may influence effect estimates. Analyses will be conducted by type of chronic disease outcome (cardiovascular disease, type 2 diabetes, cancer, chronic kidney disease, and respiratory disease) to address potential disease-specific relationships with water intake.

Further subgroup analyses will explore differences based on method of water intake assessment (self-reported vs. directly measured) to evaluate the impact of measurement bias. Participant demographics such as age and sex will be considered independently given their influence on hydration needs and chronic disease susceptibility. Geographic or climatic region will also be examined to account for environmental factors affecting hydration requirements and disease patterns. Lastly, baseline health status and presence of comorbidities will be analysed to explore potential confounding or effect modification.

In addition, sensitivity analyses will be performed to assess the robustness of the findings by excluding studies with missing or unclear data, and those assessed as having high risk of bias. Additional sensitivity analyses will examine the impact of confounder adjustment within studies. This dual approach accounts for variations in methodology and enhances clarity in synthesising study findings.

To minimise confounding, the search syntax (refer to Additional File 1) has been refined to exclude studies focusing solely on extraneous variables without addressing water intake as a primary exposure. Nonetheless, the review acknowledges the potential for residual confounding from lifestyle and environmental factors. Data will be extracted on predefined confounders, including dietary intake, physical activity, climate or geographical region, age, sex, socioeconomic status, and comorbidities. Each included study will be evaluated on whether and how these confounders were identified and adjusted for in their analyses. This information will inform the risk of bias assessment and synthesis strategy.

Where data permits, meta-regression will be conducted to evaluate the influence of confounders on pooled effect estimates. Where meta-regressions are not feasible, subgroup analyses will be used instead. While several confounders overlap with the predefined subgroup variables, their role as confounders refers to whether the original studies appropriately controlled for these variables, whereas subgroup analyses examine between-study differences.

A DerSimonian-Laird random effects model will be used for meta-analysis to account for between-study heterogeneity. The generic inverse-variance method will be applied within subgroups to determine appropriate weighting. Heterogeneity will be assessed visually using forest plots, alongside statistical assessment using the Q-test for the presence of heterogeneity and I^2^ statistic for the magnitude. I^2^ values will be interpreted in line with the Cochrane Handbook [38] as follows: 0–40% (may not be important), 30–60% (may represent moderate heterogeneity), 50–90% (may represent moderate heterogeneity, and 75–100% (considerable heterogeneity). For this review, I^2^ values greater than 50% will be considered indicative of substantial heterogeneity and may prompt subgroup or sensitivity analyses. If I^2^ exceeds 75%, a narrative synthesis will be conducted in place of meta-analysis.

The GRADE (Grading of Recommendations, Assessment, Development and Evaluation) [40] approach will be utilised to assess the strength of the cumulative body of evidence.

The GRADE system assesses risk of bias, inconsistency, indirectness, imprecision, and publication bias to determine whether the evidence has high, moderate, low or very low certainty. A ‘summary of findings’ table will be provided detailing the outcome, number of participants, globalised effect estimates, GRADE certainty, and comments will be reported in the review.

## Discussion

This systematic review will be the first to examine the hypothesis that recommended daily water intake is associated with a decreased risk of chronic disease development. To date, available evidence remains fragmented and focuses on the role of water intake on one individual chronic disease, such as certain cancers or CVD [18, 23–25]. As a result, there is insufficient evidence to explore the relationship between water intake and chronic diseases as a collective, underscoring the novelty and necessity of this systematic review. Given the escalating global burden of chronic diseases [1–4], synthesising this body of literature has significant implications for informing public health strategies and shaping future evidence-based hydration guidelines.

We acknowledge several limitations that may affect the interpretation of our findings. First, water intake data across the included studies are likely to be derived from self-reported or observational cohort data, introducing potential for measurement error and bias. Second, publication bias may arise due to the omission of unpublished studies. Third, the restriction to English-language publications may result in language bias, particularly given the global distribution of hydration research. However, this limitation was necessary for this preliminary review to focus on studies from English-speaking contexts, enabling us to develop a clear and manageable methodological framework. This framework can be adapted and expanded in future reviews to include non-English studies, thereby broadening the evidence base.

Furthermore, there are complexities regarding the measurement of water intake as overall hydration can be attributed to sources other than plain water, which this review does not consider. While this could reduce generalisability to overall hydration behaviours, this decision was methodologically important for isolating the specific impact of plain water intake on major chronic disease risk. Our aim is to form a foundational framework that is accessible to the public, where plain water, being easily measurable and modifiable, can serve as a baseline for actionable guidelines. Including all sources of hydration would not reflect real-world feasibility for public adherence and would introduce substantial heterogeneity. Further research can build upon this review to incorporate total hydration measures and explore additive or synergistic effects of alternative hydration sources.

We also recognise that some studies may not clearly identify water sources, and for the purposes of this review, water intake will be defined as tap, bottled, and/or drinking water. Variability in the study design, water intake assessment periods, and population characteristics are anticipated, which may limit cross-study comparability. Moreover, the intake range defined in our study as adequate (2 to 3.7 l daily) is based on established guidelines [17–19, 28], but some included studies may use differing thresholds. Such discrepancies could introduce intercorrelation concerns and risk inflating pooled estimates. While efforts will be made to adjust for key confounders where data allow, we acknowledge that residual confounding may persist due to variation in how individual studies control for these. This limitation will be carefully considered during data synthesis and interpretation.

Overall, while this review is subject to methodological constrains inherent to early-stage evidence synthesis, it represents a critical step toward consolidating fragmented data on plain water intake and chronic disease risk. Findings from this review will provide a preliminary, evidence informed platform for subsequent research.

In conclusion, this protocol outlines a novel and methodologically rigorous systematic review aimed at collating existing data on the relationship between plain water intake and the risk of chronic disease. The finding are expected to make a valuable contribution to the scientific literature and offer an evidence-based foundation for future research. Ultimately, this review will support the development of clearer, more actionable hydration guidelines to improve public health outcomes.

## Supplementary Information

Additional file 1: iMedical subject headings Medline search strategy terms.

Additional file 2: Checklist item.

## Data Availability

Mesh headings will be available as a supplementary document with the final systematic review.
