# Supplementary material for: Effect of adequate daily water intake versus inadequate water intake on the risk of major chronic diseases in healthy adults: a systematic review protocol
Source: Syst Rev. 2026 Mar 4;15:125. doi: 10.1186/s13643-026-03108-x (PMC13067716; doi:10.1186/s13643-026-03108-x)
Supplement: Supplementary file 1 — Additional file 1: iMedical subject headings Medline search strategy terms. [file 13643_2026_3108_MOESM1_ESM.docx]

# iMedical Subject Headings Medline Search Strategy Terms

**Translations**

**fluid:** "fluid"[All Fields] OR "fluid's"[All Fields] OR "fluids"[All Fields]

**intake:** "intake"[All Fields] OR "intake's"[All Fields] OR "intakes"[All Fields]

**drinking water:** "drinking water"[MeSH Terms] OR ("drinking"[All Fields] AND "water"[All Fields]) OR "drinking water"[All Fields]

**bottle:** "bottle"[All Fields] OR "bottle's"[All Fields] OR "bottled"[All Fields] OR "bottles"[All Fields] OR "bottling"[All Fields]

**water:** "water"[MeSH Terms] OR "water"[All Fields] OR "water's"[All Fields] OR "watered"[All Fields] OR "waterer"[All Fields] OR "waterers"[All Fields] OR "watering"[All Fields] OR "waterings"[All Fields] OR "waters"[All Fields]

**water:** "water"[MeSH Terms] OR "water"[All Fields] OR "water's"[All Fields] OR "watered"[All Fields] OR "waterer"[All Fields] OR "waterers"[All Fields] OR "watering"[All Fields] OR "waterings"[All Fields] OR "waters"[All Fields]

**water:** "water"[MeSH Terms] OR "water"[All Fields] OR "water's"[All Fields] OR "watered"[All Fields] OR "waterer"[All Fields] OR "waterers"[All Fields] OR "watering"[All Fields] OR "waterings"[All Fields] OR "waters"[All Fields]

**plain:** "plain"[All Fields] OR "plains"[All Fields]

**water:** "water"[MeSH Terms] OR "water"[All Fields] OR "water's"[All Fields] OR "watered"[All Fields] OR "waterer"[All Fields] OR "waterers"[All Fields] OR "watering"[All Fields] OR "waterings"[All Fields] OR "waters"[All Fields]

**water consumption:** "drinking"[MeSH Terms] OR "drinking"[All Fields] OR ("water"[All Fields] AND "consumption"[All Fields]) OR "water consumption"[All Fields]

**water intake:** "drinking"[MeSH Terms] OR "drinking"[All Fields] OR ("water"[All Fields] AND "intake"[All Fields]) OR "water intake"[All Fields]

**consume:** "consumable"[All Fields] OR "consumables"[All Fields] OR "consume"[All Fields] OR "consumed"[All Fields] OR "consumer"[All Fields] OR "consumer's"[All Fields] OR "consumers"[All Fields] OR "consumes"[All Fields] OR "consuming"[All Fields]

**intake:** "intake"[All Fields] OR "intake's"[All Fields] OR "intakes"[All Fields]

**drink:** "drink"[All Fields] OR "drinking"[MeSH Terms] OR "drinking"[All Fields] OR "alcohol drinking"[MeSH Terms] OR ("alcohol"[All Fields] AND "drinking"[All Fields]) OR "alcohol drinking"[All Fields] OR "drinkings"[All Fields] OR "drinking's"[All Fields] OR "drinks"[All Fields]

**metastases:** "metastasation"[All Fields] OR "metastasic"[All Fields] OR "metastasing"[All Fields] OR "metastasise"[All Fields] OR "metastasised"[All Fields] OR "metastasises"[All Fields] OR "metastasising"[All Fields] OR "metastasization"[All Fields] OR "metastasizes"[All Fields] OR "metastasizing"[All Fields] OR "neoplasm metastasis"[MeSH Terms] OR ("neoplasm"[All Fields] AND "metastasis"[All Fields]) OR "neoplasm metastasis"[All Fields] OR "metastase"[All Fields] OR "metastases"[All Fields] OR "metastasize"[All Fields] OR "metastasized"[All Fields]

**metastasis:** "metastasi"[All Fields] OR "neoplasm metastasis"[MeSH Terms] OR ("neoplasm"[All Fields] AND "metastasis"[All Fields]) OR "neoplasm metastasis"[All Fields] OR "metastasis"[All Fields]

**melanoma:** "melanoma"[MeSH Terms] OR "melanoma"[All Fields] OR "melanomas"[All Fields] OR "melanoma's"[All Fields]

**blastoma:** "blastomas"[All Fields] OR "neoplasms"[MeSH Terms] OR "neoplasms"[All Fields] OR "blastoma"[All Fields]

**malignant:** "malign"[All Fields] OR "malignance"[All Fields] OR "malignances"[All Fields] OR "malignant"[All Fields] OR "malignants"[All Fields] OR "malignities"[All Fields] OR "malignity"[All Fields] OR "malignization"[All Fields] OR "malignized"[All Fields] OR "maligns"[All Fields] OR "neoplasms"[MeSH Terms] OR "neoplasms"[All Fields] OR "malignancies"[All Fields] OR "malignancy"[All Fields]

**malignancy:** "malign"[All Fields] OR "malignance"[All Fields] OR "malignances"[All Fields] OR "malignant"[All Fields] OR "malignants"[All Fields] OR "malignities"[All Fields] OR "malignity"[All Fields] OR "malignization"[All Fields] OR "malignized"[All Fields] OR "maligns"[All Fields] OR "neoplasms"[MeSH Terms] OR "neoplasms"[All Fields] OR "malignancies"[All Fields] OR "malignancy"[All Fields]

**leukaemia:** "leukaemia"[All Fields] OR "leukemia"[MeSH Terms] OR "leukemia"[All Fields] OR "leukaemias"[All Fields] OR "leukemias"[All Fields] OR "leukemia's"[All Fields]

**adenocarcinoma:** "adenocarcinoma"[MeSH Terms] OR "adenocarcinoma"[All Fields] OR "adenocarcinomas"[All Fields] OR "adenocarcinoma's"[All Fields]

**lymphoma:** "lymphoma"[MeSH Terms] OR "lymphoma"[All Fields] OR "lymphomas"[All Fields] OR "lymphoma's"[All Fields]

**sarcoma:** "sarcoma"[MeSH Terms] OR "sarcoma"[All Fields] OR "sarcomas"[All Fields] OR "sarcoma's"[All Fields]

**carcinoma:** "carcinoma"[MeSH Terms] OR "carcinoma"[All Fields] OR "carcinomas"[All Fields] OR "carcinoma's"[All Fields]

**tumour:** "cysts"[MeSH Terms] OR "cysts"[All Fields] OR "cyst"[All Fields] OR "neurofibroma"[MeSH Terms] OR "neurofibroma"[All Fields] OR "neurofibromas"[All Fields] OR "tumor's"[All Fields] OR "tumoral"[All Fields] OR "tumorous"[All Fields] OR "tumour"[All Fields] OR "neoplasms"[MeSH Terms] OR "neoplasms"[All Fields] OR "tumor"[All Fields] OR "tumour's"[All Fields] OR "tumoural"[All Fields] OR "tumourous"[All Fields] OR "tumours"[All Fields] OR "tumors"[All Fields]

**cancer:** "cancer's"[All Fields] OR "cancerated"[All Fields] OR "canceration"[All Fields] OR "cancerization"[All Fields] OR "cancerized"[All Fields] OR "cancerous"[All Fields] OR "neoplasms"[MeSH Terms] OR "neoplasms"[All Fields] OR "cancer"[All Fields] OR "cancers"[All Fields]

**neoplasm:** "neoplasm's"[All Fields] OR "neoplasms"[MeSH Terms] OR "neoplasms"[All Fields] OR "neoplasm"[All Fields]

**neoplasms:** "neoplasm's"[All Fields] OR "neoplasms"[MeSH Terms] OR "neoplasms"[All Fields] OR "neoplasm"[All Fields]

**Cardiovascular Diseases:** "cardiovascular diseases"[MeSH Terms] OR ("cardiovascular"[All Fields] AND "diseases"[All Fields]) OR "cardiovascular diseases"[All Fields]

**blood pressure:** "blood pressure"[MeSH Terms] OR ("blood"[All Fields] AND "pressure"[All Fields]) OR "blood pressure"[All Fields] OR "blood pressure determination"[MeSH Terms] OR ("blood"[All Fields] AND "pressure"[All Fields] AND "determination"[All Fields]) OR "blood pressure determination"[All Fields] OR "arterial pressure"[MeSH Terms] OR ("arterial"[All Fields] AND "pressure"[All Fields]) OR "arterial pressure"[All Fields]

**ischaemic heart disease:** "ischaemic heart disease"[All Fields] OR "myocardial ischemia"[MeSH Terms] OR ("myocardial"[All Fields] AND "ischemia"[All Fields]) OR "myocardial ischemia"[All Fields] OR ("ischemic"[All Fields] AND "heart"[All Fields] AND "disease"[All Fields]) OR "ischemic heart disease"[All Fields] OR "coronary artery disease"[MeSH Terms] OR ("coronary"[All Fields] AND "artery"[All Fields] AND "disease"[All Fields]) OR "coronary artery disease"[All Fields]

**Myocardial Infarction:** "myocardial infarction"[MeSH Terms] OR ("myocardial"[All Fields] AND "infarction"[All Fields]) OR "myocardial infarction"[All Fields]

**event:** "event"[All Fields] OR "event's"[All Fields] OR "events"[All Fields]

**infarction:** "infarctation"[All Fields] OR "infarcted"[All Fields] OR "infarctic"[All Fields] OR "infarcting"[All Fields] OR "infarction"[MeSH Terms] OR "infarction"[All Fields] OR "infarct"[All Fields] OR "infarctions"[All Fields] OR "infarcts"[All Fields] OR "infarctive"[All Fields]

**infarct:** "infarctation"[All Fields] OR "infarcted"[All Fields] OR "infarctic"[All Fields] OR "infarcting"[All Fields] OR "infarction"[MeSH Terms] OR "infarction"[All Fields] OR "infarct"[All Fields] OR "infarctions"[All Fields] OR "infarcts"[All Fields] OR "infarctive"[All Fields]

**failure:** "failure"[All Fields] OR "failures"[All Fields]

**attack:** "attack"[All Fields] OR "attacked"[All Fields] OR "attacker"[All Fields] OR "attacker's"[All Fields] OR "attackers"[All Fields] OR "attacking"[All Fields] OR "attacks"[All Fields]

**arrest:** "arrestant"[All Fields] OR "arrestants"[All Fields] OR "arresting"[All Fields] OR "arrestment"[All Fields] OR "arrests"[All Fields] OR "heart arrest"[MeSH Terms] OR ("heart"[All Fields] AND "arrest"[All Fields]) OR "heart arrest"[All Fields] OR "arrest"[All Fields] OR "arrested"[All Fields]

**disorder:** "disease"[MeSH Terms] OR "disease"[All Fields] OR "disorder"[All Fields] OR "disorders"[All Fields] OR "disorder's"[All Fields] OR "disordes"[All Fields]

**disease:** "disease"[MeSH Terms] OR "disease"[All Fields] OR "diseases"[All Fields] OR "disease's"[All Fields] OR "diseased"[All Fields]

**myocardia:** "myocardium"[MeSH Terms] OR "myocardium"[All Fields] OR "myocardia"[All Fields]

**cardiac:** "cardiacs"[All Fields] OR "heart"[MeSH Terms] OR "heart"[All Fields] OR "cardiac"[All Fields]

**artery:** "arterialization"[All Fields] OR "arterializations"[All Fields] OR "arterialize"[All Fields] OR "arterialized"[All Fields] OR "arterializing"[All Fields] OR "arterially"[All Fields] OR "arterials"[All Fields] OR "arterie"[All Fields] OR "arteries"[MeSH Terms] OR "arteries"[All Fields] OR "arterial"[All Fields] OR "arteris"[All Fields] OR "artery"[All Fields] OR "arterious"[All Fields] OR "artery's"[All Fields] OR "arterys"[All Fields]

**heart:** "heart"[MeSH Terms] OR "heart"[All Fields] OR "hearts"[All Fields] OR "heart's"[All Fields]

**ischaemia:** "ischaemia"[All Fields] OR "ischemia"[MeSH Terms] OR "ischemia"[All Fields] OR "ischaemias"[All Fields] OR "ischemias"[All Fields]

**coronary:** "coronaries"[All Fields] OR "heart"[MeSH Terms] OR "heart"[All Fields] OR "coronary"[All Fields]

**heart diseases:** "heart diseases"[MeSH Terms] OR ("heart"[All Fields] AND "diseases"[All Fields]) OR "heart diseases"[All Fields]

**heart disease:** "heart diseases"[MeSH Terms] OR ("heart"[All Fields] AND "diseases"[All Fields]) OR "heart diseases"[All Fields] OR ("heart"[All Fields] AND "disease"[All Fields]) OR "heart disease"[All Fields]

**hypertension:** "hypertense"[All Fields] OR "hypertension"[MeSH Terms] OR "hypertension"[All Fields] OR "hypertension's"[All Fields] OR "hypertensions"[All Fields] OR "hypertensive"[All Fields] OR "hypertensive's"[All Fields] OR "hypertensives"[All Fields]

**CHD:** "Cancer Health Disparities"[Journal:__jid101728460] OR "chd"[All Fields]

**Cardiovascular Disease:** "cardiovascular diseases"[MeSH Terms] OR ("cardiovascular"[All Fields] AND "diseases"[All Fields]) OR "cardiovascular diseases"[All Fields] OR ("cardiovascular"[All Fields] AND "disease"[All Fields]) OR "cardiovascular disease"[All Fields]

**Cardiovascular Diseases:** "cardiovascular diseases"[MeSH Terms] OR ("cardiovascular"[All Fields] AND "diseases"[All Fields]) OR "cardiovascular diseases"[All Fields]

**pneumonitis:** "pneumonia"[MeSH Terms] OR "pneumonia"[All Fields] OR "pneumonitis"[All Fields]

**inspiratory:** "inhalation"[MeSH Terms] OR "inhalation"[All Fields] OR "inspiratory"[All Fields]

**expiratory:** "exhalation"[MeSH Terms] OR "exhalation"[All Fields] OR "expiratory"[All Fields]

**wheezing:** "respiratory sounds"[MeSH Terms] OR ("respiratory"[All Fields] AND "sounds"[All Fields]) OR "respiratory sounds"[All Fields] OR "wheeze"[All Fields] OR "wheezes"[All Fields] OR "wheezing"[All Fields] OR "wheezed"[All Fields]

**wheezes:** "respiratory sounds"[MeSH Terms] OR ("respiratory"[All Fields] AND "sounds"[All Fields]) OR "respiratory sounds"[All Fields] OR "wheeze"[All Fields] OR "wheezes"[All Fields] OR "wheezing"[All Fields] OR "wheezed"[All Fields]

**wheeze:** "respiratory sounds"[MeSH Terms] OR ("respiratory"[All Fields] AND "sounds"[All Fields]) OR "respiratory sounds"[All Fields] OR "wheeze"[All Fields] OR "wheezes"[All Fields] OR "wheezing"[All Fields] OR "wheezed"[All Fields]

**dyspnoea:** "dyspneas"[All Fields] OR "dyspnoea"[All Fields] OR "dyspnea"[MeSH Terms] OR "dyspnea"[All Fields]

**difficult breathing:** "dyspnea"[MeSH Terms] OR "dyspnea"[All Fields] OR ("difficult"[All Fields] AND "breathing"[All Fields]) OR "difficult breathing"[All Fields]

**breathing difficulties:** "dyspnea"[MeSH Terms] OR "dyspnea"[All Fields] OR ("breathing"[All Fields] AND "difficulties"[All Fields]) OR "breathing difficulties"[All Fields]

**breathing difficulty:** "dyspnea"[MeSH Terms] OR "dyspnea"[All Fields] OR ("breathing"[All Fields] AND "difficulty"[All Fields]) OR "breathing difficulty"[All Fields]

**breathing:** "breath"[All Fields] OR "breathe"[All Fields] OR "breathed"[All Fields] OR "breathes"[All Fields] OR "breathings"[All Fields] OR "breaths"[All Fields] OR "respiration"[MeSH Terms] OR "respiration"[All Fields] OR "breathing"[All Fields]

**sputum:** "sputum"[MeSH Terms] OR "sputum"[All Fields] OR "sputums"[All Fields]

**cough:** "cough"[MeSH Terms] OR "cough"[All Fields] OR "coughing"[All Fields] OR "coughs"[All Fields] OR "coughed"[All Fields]

**fibrosis:** "fibrosi"[All Fields] OR "fibrosing"[All Fields] OR "fibrosis"[MeSH Terms] OR "fibrosis"[All Fields] OR "fibrose"[All Fields] OR "fibroses"[All Fields]

**asthma:** "asthma"[MeSH Terms] OR "asthma"[All Fields] OR "asthmas"[All Fields] OR "asthma's"[All Fields]

**chronic obstructive pulmonary disease:** "pulmonary disease, chronic obstructive"[MeSH Terms] OR ("pulmonary"[All Fields] AND "disease"[All Fields] AND "chronic"[All Fields] AND "obstructive"[All Fields]) OR "chronic obstructive pulmonary disease"[All Fields] OR ("chronic"[All Fields] AND "obstructive"[All Fields] AND "pulmonary"[All Fields] AND "disease"[All Fields])

**COPD:** "pulmonary disease, chronic obstructive"[MeSH Terms] OR ("pulmonary"[All Fields] AND "disease"[All Fields] AND "chronic"[All Fields] AND "obstructive"[All Fields]) OR "chronic obstructive pulmonary disease"[All Fields] OR "copd"[All Fields]

**lungs:** "lung"[MeSH Terms] OR "lung"[All Fields] OR "lungs"[All Fields] OR "lung's"[All Fields]

**pulmonary:** "lung"[MeSH Terms] OR "lung"[All Fields] OR "pulmonary"[All Fields]

**respiratory:** "Eur Med J Respir"[Journal:__jid101776214] OR "respiratory"[All Fields]

**impaired:** "impair"[All Fields] OR "impaired"[All Fields] OR "impairement"[All Fields] OR "impairements"[All Fields] OR "impairing"[All Fields] OR "impairment"[All Fields] OR "impairments"[All Fields] OR "impairs"[All Fields]

**fasting:** "fasted"[All Fields] OR "fasting"[MeSH Terms] OR "fasting"[All Fields] OR "fastings"[All Fields] OR "fasts"[All Fields]

**glucose:** "glucose"[MeSH Terms] OR "glucose"[All Fields] OR "glucoses"[All Fields] OR "glucose's"[All Fields]

**impaired glucose tolerance:** "glucose intolerance"[MeSH Terms] OR ("glucose"[All Fields] AND "intolerance"[All Fields]) OR "glucose intolerance"[All Fields] OR ("impaired"[All Fields] AND "glucose"[All Fields] AND "tolerance"[All Fields]) OR "impaired glucose tolerance"[All Fields]

**impaired:** "impair"[All Fields] OR "impaired"[All Fields] OR "impairement"[All Fields] OR "impairements"[All Fields] OR "impairing"[All Fields] OR "impairment"[All Fields] OR "impairments"[All Fields] OR "impairs"[All Fields]

**glucose:** "glucose"[MeSH Terms] OR "glucose"[All Fields] OR "glucoses"[All Fields] OR "glucose's"[All Fields]

**Glucose:** "glucose"[MeSH Terms] OR "glucose"[All Fields] OR "glucoses"[All Fields] OR "glucose's"[All Fields]

**Intolerant:** "intolerabilities"[All Fields] OR "intolerability"[All Fields] OR "intolerable"[All Fields] OR "intolerably"[All Fields] OR "intolerance"[All Fields] OR "intolerances"[All Fields] OR "intolerant"[All Fields] OR "intolerants"[All Fields]

**Glucose Intolerance:** "glucose intolerance"[MeSH Terms] OR ("glucose"[All Fields] AND "intolerance"[All Fields]) OR "glucose intolerance"[All Fields]

**hyperglycaemia:** "hyperglycaemia"[All Fields] OR "hyperglycemia"[MeSH Terms] OR "hyperglycemia"[All Fields] OR "hyperglycaemias"[All Fields] OR "hyperglycemias"[All Fields] OR "hyperglycemia's"[All Fields]

**insulin:** "insulin"[MeSH Terms] OR "insulin"[All Fields] OR "insulin's"[All Fields] OR "insuline"[All Fields] OR "insulinic"[All Fields] OR "insulinization"[All Fields] OR "insulinized"[All Fields] OR "insulins"[MeSH Terms] OR "insulins"[All Fields]

**NIDDM:** "diabetes mellitus, type 2"[MeSH Terms] OR "type 2 diabetes mellitus"[All Fields] OR "niddm"[All Fields] OR "niddms"[All Fields]

**type 2 diabetes mellitus:** "diabetes mellitus, type 2"[MeSH Terms] OR "type 2 diabetes mellitus"[All Fields]

**Diabetes Mellitus:** "diabetes mellitus"[MeSH Terms] OR ("diabetes"[All Fields] AND "mellitus"[All Fields]) OR "diabetes mellitus"[All Fields]

**decrease:** "decrease"[All Fields] OR "decreased"[All Fields] OR "decreases"[All Fields] OR "decreasing"[All Fields]

**glomerular filtration rate:** "glomerular filtration rate"[MeSH Terms] OR ("glomerular"[All Fields] AND "filtration"[All Fields] AND "rate"[All Fields]) OR "glomerular filtration rate"[All Fields]

**glomerular:** "kidney glomerulus"[MeSH Terms] OR ("kidney"[All Fields] AND "glomerulus"[All Fields]) OR "kidney glomerulus"[All Fields] OR "glomerular"[All Fields]

**filtration:** "filtrate"[All Fields] OR "filtrated"[All Fields] OR "filtrates"[All Fields] OR "filtrating"[All Fields] OR "filtration"[MeSH Terms] OR "filtration"[All Fields] OR "filtrations"[All Fields]

**kidney diseases:** "kidney diseases"[MeSH Terms] OR ("kidney"[All Fields] AND "diseases"[All Fields]) OR "kidney diseases"[All Fields]

**polycystic kidney disease:** "polycystic kidney diseases"[MeSH Terms] OR ("polycystic"[All Fields] AND "kidney"[All Fields] AND "diseases"[All Fields]) OR "polycystic kidney diseases"[All Fields] OR ("polycystic"[All Fields] AND "kidney"[All Fields] AND "disease"[All Fields]) OR "polycystic kidney disease"[All Fields]

**kidney disease:** "kidney diseases"[MeSH Terms] OR ("kidney"[All Fields] AND "diseases"[All Fields]) OR "kidney diseases"[All Fields] OR ("kidney"[All Fields] AND "disease"[All Fields]) OR "kidney disease"[All Fields]

**kidney:** "kidney"[MeSH Terms] OR "kidney"[All Fields] OR "kidneys"[All Fields] OR "kidney's"[All Fields]

**chronic kidney disease:** "renal insufficiency, chronic"[MeSH Terms] OR ("renal"[All Fields] AND "insufficiency"[All Fields] AND "chronic"[All Fields]) OR "chronic renal insufficiency"[All Fields] OR ("chronic"[All Fields] AND "kidney"[All Fields] AND "disease"[All Fields]) OR "chronic kidney disease"[All Fields]

**Full Search Syntax:**

(((("fluid"[All Fields] OR "fluid s"[All Fields] OR "fluids"[All Fields]) AND ("intake"[All Fields] OR "intake s"[All Fields] OR "intakes"[All Fields])) OR ("drinking water"[MeSH Terms] OR ("drinking"[All Fields] AND "water"[All Fields]) OR "drinking water"[All Fields]) OR (("bottle"[All Fields] OR "bottle s"[All Fields] OR "bottled"[All Fields] OR "bottles"[All Fields] OR "bottling"[All Fields]) AND ("water"[MeSH Terms] OR "water"[All Fields] OR "water s"[All Fields] OR "watered"[All Fields] OR "waterer"[All Fields] OR "waterers"[All Fields] OR "watering"[All Fields] OR "waterings"[All Fields] OR "waters"[All Fields])) OR ("tap"[All Fields] AND ("water"[MeSH Terms] OR "water"[All Fields] OR "water s"[All Fields] OR "watered"[All Fields] OR "waterer"[All Fields] OR "waterers"[All Fields] OR "watering"[All Fields] OR "waterings"[All Fields] OR "waters"[All Fields])) OR ("water"[MeSH Terms] OR "water"[All Fields] OR "water s"[All Fields] OR "watered"[All Fields] OR "waterer"[All Fields] OR "waterers"[All Fields] OR "watering"[All Fields] OR "waterings"[All Fields] OR "waters"[All Fields] OR (("plain"[All Fields] OR "plains"[All Fields]) AND ("water"[MeSH Terms] OR "water"[All Fields] OR "water s"[All Fields] OR "watered"[All Fields] OR "waterer"[All Fields] OR "waterers"[All Fields] OR "watering"[All Fields] OR "waterings"[All Fields] OR "waters"[All Fields]))) OR ("drinking"[MeSH Terms] OR "drinking"[All Fields] OR ("water"[All Fields] AND "consumption"[All Fields]) OR "water consumption"[All Fields]) OR ("drinking"[MeSH Terms] OR "drinking"[All Fields] OR ("water"[All Fields] AND "intake"[All Fields]) OR "water intake"[All Fields])) AND ("consumable"[All Fields] OR "consumables"[All Fields] OR "consume"[All Fields] OR "consumed"[All Fields] OR "consumer"[All Fields] OR "consumer s"[All Fields] OR "consumers"[All Fields] OR "consumes"[All Fields] OR "consuming"[All Fields] OR ("intake"[All Fields] OR "intake s"[All Fields] OR "intakes"[All Fields]) OR ("drink"[All Fields] OR "drinking"[MeSH Terms] OR "drinking"[All Fields] OR "alcohol drinking"[MeSH Terms] OR ("alcohol"[All Fields] AND "drinking"[All Fields]) OR "alcohol drinking"[All Fields] OR "drinkings"[All Fields] OR "drinks"[All Fields]) OR "drank"[All Fields]) AND ("metastasation"[All Fields] OR "metastasic"[All Fields] OR "metastasing"[All Fields] OR "metastasise"[All Fields] OR "metastasised"[All Fields] OR "metastasises"[All Fields] OR "metastasising"[All Fields] OR "metastasization"[All Fields] OR "metastasizes"[All Fields] OR "metastasizing"[All Fields] OR "neoplasm metastasis"[MeSH Terms] OR ("neoplasm"[All Fields] AND "metastasis"[All Fields]) OR "neoplasm metastasis"[All Fields] OR "metastase"[All Fields] OR "metastases"[All Fields] OR "metastasize"[All Fields] OR "metastasized"[All Fields] OR ("metastasi"[All Fields] OR "neoplasm metastasis"[MeSH Terms] OR ("neoplasm"[All Fields] AND "metastasis"[All Fields]) OR "neoplasm metastasis"[All Fields] OR "metastasis"[All Fields]) OR ("melanoma"[MeSH Terms] OR "melanoma"[All Fields] OR "melanomas"[All Fields] OR "melanoma s"[All Fields]) OR ("blastomas"[All Fields] OR "neoplasms"[MeSH Terms] OR "neoplasms"[All Fields] OR "blastoma"[All Fields]) OR ("malign"[All Fields] OR "malignance"[All Fields] OR "malignances"[All Fields] OR "malignant"[All Fields] OR "malignants"[All Fields] OR "malignities"[All Fields] OR "malignity"[All Fields] OR "malignization"[All Fields] OR "malignized"[All Fields] OR "maligns"[All Fields] OR "neoplasms"[MeSH Terms] OR "neoplasms"[All Fields] OR "malignancies"[All Fields] OR "malignancy"[All Fields]) OR ("malign"[All Fields] OR "malignance"[All Fields] OR "malignances"[All Fields] OR "malignant"[All Fields] OR "malignants"[All Fields] OR "malignities"[All Fields] OR "malignity"[All Fields] OR "malignization"[All Fields] OR "malignized"[All Fields] OR "maligns"[All Fields] OR "neoplasms"[MeSH Terms] OR "neoplasms"[All Fields] OR "malignancies"[All Fields] OR "malignancy"[All Fields]) OR ("leukaemia"[All Fields] OR "leukemia"[MeSH Terms] OR "leukemia"[All Fields] OR "leukaemias"[All Fields] OR "leukemias"[All Fields] OR "leukemia s"[All Fields]) OR ("adenocarcinoma"[MeSH Terms] OR "adenocarcinoma"[All Fields] OR "adenocarcinomas"[All Fields] OR "adenocarcinoma s"[All Fields]) OR ("lymphoma"[MeSH Terms] OR "lymphoma"[All Fields] OR "lymphomas"[All Fields] OR "lymphoma s"[All Fields]) OR ("sarcoma"[MeSH Terms] OR "sarcoma"[All Fields] OR "sarcomas"[All Fields] OR "sarcoma s"[All Fields]) OR ("carcinoma"[MeSH Terms] OR "carcinoma"[All Fields] OR "carcinomas"[All Fields] OR "carcinoma s"[All Fields]) OR ("cysts"[MeSH Terms] OR "cysts"[All Fields] OR "cyst"[All Fields] OR "neurofibroma"[MeSH Terms] OR "neurofibroma"[All Fields] OR "neurofibromas"[All Fields] OR "tumor s"[All Fields] OR "tumoral"[All Fields] OR "tumorous"[All Fields] OR "tumour"[All Fields] OR "neoplasms"[MeSH Terms] OR "neoplasms"[All Fields] OR "tumor"[All Fields] OR "tumour s"[All Fields] OR "tumoural"[All Fields] OR "tumourous"[All Fields] OR "tumours"[All Fields] OR "tumors"[All Fields]) OR ("cancer s"[All Fields] OR "cancerated"[All Fields] OR "canceration"[All Fields] OR "cancerization"[All Fields] OR "cancerized"[All Fields] OR "cancerous"[All Fields] OR "neoplasms"[MeSH Terms] OR "neoplasms"[All Fields] OR "cancer"[All Fields] OR "cancers"[All Fields]) OR ("neoplasm s"[All Fields] OR "neoplasms"[MeSH Terms] OR "neoplasms"[All Fields] OR "neoplasm"[All Fields]) OR ("neoplasm s"[All Fields] OR "neoplasms"[MeSH Terms] OR "neoplasms"[All Fields] OR "neoplasm"[All Fields]) OR ("cardiovascular diseases"[MeSH Terms] OR ("cardiovascular"[All Fields] AND "diseases"[All Fields]) OR "cardiovascular diseases"[All Fields] OR ("blood pressure"[MeSH Terms] OR ("blood"[All Fields] AND "pressure"[All Fields]) OR "blood pressure"[All Fields] OR "blood pressure determination"[MeSH Terms] OR ("blood"[All Fields] AND "pressure"[All Fields] AND "determination"[All Fields]) OR "blood pressure determination"[All Fields] OR "arterial pressure"[MeSH Terms] OR ("arterial"[All Fields] AND "pressure"[All Fields]) OR "arterial pressure"[All Fields]) OR ("ischaemic heart disease"[All Fields] OR "myocardial ischemia"[MeSH Terms] OR ("myocardial"[All Fields] AND "ischemia"[All Fields]) OR "myocardial ischemia"[All Fields] OR ("ischemic"[All Fields] AND "heart"[All Fields] AND "disease"[All Fields]) OR "ischemic heart disease"[All Fields] OR "coronary artery disease"[MeSH Terms] OR ("coronary"[All Fields] AND "artery"[All Fields] AND "disease"[All Fields]) OR "coronary artery disease"[All Fields]) OR ("myocardial infarction"[MeSH Terms] OR ("myocardial"[All Fields] AND "infarction"[All Fields]) OR "myocardial infarction"[All Fields]) OR ("event"[All Fields] OR "event s"[All Fields] OR "events"[All Fields]) OR ("infarctation"[All Fields] OR "infarcted"[All Fields] OR "infarctic"[All Fields] OR "infarcting"[All Fields] OR "infarction"[MeSH Terms] OR "infarction"[All Fields] OR "infarct"[All Fields] OR "infarctions"[All Fields] OR "infarcts"[All Fields] OR "infarctive"[All Fields]) OR ("infarctation"[All Fields] OR "infarcted"[All Fields] OR "infarctic"[All Fields] OR "infarcting"[All Fields] OR "infarction"[MeSH Terms] OR "infarction"[All Fields] OR "infarct"[All Fields] OR "infarctions"[All Fields] OR "infarcts"[All Fields] OR "infarctive"[All Fields]) OR ("failure"[All Fields] OR "failures"[All Fields]) OR ("attack"[All Fields] OR "attacked"[All Fields] OR "attacker"[All Fields] OR "attacker s"[All Fields] OR "attackers"[All Fields] OR "attacking"[All Fields] OR "attacks"[All Fields]) OR ("arrestant"[All Fields] OR "arrestants"[All Fields] OR "arresting"[All Fields] OR "arrestment"[All Fields] OR "arrests"[All Fields] OR "heart arrest"[MeSH Terms] OR ("heart"[All Fields] AND "arrest"[All Fields]) OR "heart arrest"[All Fields] OR "arrest"[All Fields] OR "arrested"[All Fields]) OR ("disease"[MeSH Terms] OR "disease"[All Fields] OR "disorder"[All Fields] OR "disorders"[All Fields] OR "disorder s"[All Fields] OR "disordes"[All Fields]) OR ("disease"[MeSH Terms] OR "disease"[All Fields] OR "diseases"[All Fields] OR "disease s"[All Fields] OR "diseased"[All Fields]) OR ("myocardium"[MeSH Terms] OR "myocardium"[All Fields] OR "myocardia"[All Fields]) OR ("cardiacs"[All Fields] OR "heart"[MeSH Terms] OR "heart"[All Fields] OR "cardiac"[All Fields]) OR ("arterialization"[All Fields] OR "arterializations"[All Fields] OR "arterialize"[All Fields] OR "arterialized"[All Fields] OR "arterializing"[All Fields] OR "arterially"[All Fields] OR "arterials"[All Fields] OR "arterie"[All Fields] OR "arteries"[MeSH Terms] OR "arteries"[All Fields] OR "arterial"[All Fields] OR "arteris"[All Fields] OR "artery"[All Fields] OR "arterious"[All Fields] OR "artery s"[All Fields] OR "arterys"[All Fields]) OR ("heart"[MeSH Terms] OR "heart"[All Fields] OR "hearts"[All Fields] OR "heart s"[All Fields]) OR ("ischaemia"[All Fields] OR "ischemia"[MeSH Terms] OR "ischemia"[All Fields] OR "ischaemias"[All Fields] OR "ischemias"[All Fields]) OR ("coronaries"[All Fields] OR "heart"[MeSH Terms] OR "heart"[All Fields] OR "coronary"[All Fields]) OR ("heart diseases"[MeSH Terms] OR ("heart"[All Fields] AND "diseases"[All Fields]) OR "heart diseases"[All Fields]) OR ("heart diseases"[MeSH Terms] OR ("heart"[All Fields] AND "diseases"[All Fields]) OR "heart diseases"[All Fields] OR ("heart"[All Fields] AND "disease"[All Fields]) OR "heart disease"[All Fields]) OR "HTN"[All Fields] OR ("hypertense"[All Fields] OR "hypertension"[MeSH Terms] OR "hypertension"[All Fields] OR "hypertension s"[All Fields] OR "hypertensions"[All Fields] OR "hypertensive"[All Fields] OR "hypertensive s"[All Fields] OR "hypertensives"[All Fields]) OR ("cancer health disparities"[Journal] OR "chd"[All Fields]) OR "CVD"[All Fields] OR ("cardiovascular diseases"[MeSH Terms] OR ("cardiovascular"[All Fields] AND "diseases"[All Fields]) OR "cardiovascular diseases"[All Fields] OR ("cardiovascular"[All Fields] AND "disease"[All Fields]) OR "cardiovascular disease"[All Fields]) OR ("cardiovascular diseases"[MeSH Terms] OR ("cardiovascular"[All Fields] AND "diseases"[All Fields]) OR "cardiovascular diseases"[All Fields])) OR ("pneumonia"[MeSH Terms] OR "pneumonia"[All Fields] OR "pneumonitis"[All Fields] OR ("inhalation"[MeSH Terms] OR "inhalation"[All Fields] OR "inspiratory"[All Fields]) OR ("exhalation"[MeSH Terms] OR "exhalation"[All Fields] OR "expiratory"[All Fields]) OR ("respiratory sounds"[MeSH Terms] OR ("respiratory"[All Fields] AND "sounds"[All Fields]) OR "respiratory sounds"[All Fields] OR "wheeze"[All Fields] OR "wheezes"[All Fields] OR "wheezing"[All Fields] OR "wheezed"[All Fields]) OR ("respiratory sounds"[MeSH Terms] OR ("respiratory"[All Fields] AND "sounds"[All Fields]) OR "respiratory sounds"[All Fields] OR "wheeze"[All Fields] OR "wheezes"[All Fields] OR "wheezing"[All Fields] OR "wheezed"[All Fields]) OR ("respiratory sounds"[MeSH Terms] OR ("respiratory"[All Fields] AND "sounds"[All Fields]) OR "respiratory sounds"[All Fields] OR "wheeze"[All Fields] OR "wheezes"[All Fields] OR "wheezing"[All Fields] OR "wheezed"[All Fields]) OR ("dyspneas"[All Fields] OR "dyspnoea"[All Fields] OR "dyspnea"[MeSH Terms] OR "dyspnea"[All Fields]) OR ("dyspnea"[MeSH Terms] OR "dyspnea"[All Fields] OR ("difficult"[All Fields] AND "breathing"[All Fields]) OR "difficult breathing"[All Fields]) OR ("dyspnea"[MeSH Terms] OR "dyspnea"[All Fields] OR ("breathing"[All Fields] AND "difficulties"[All Fields]) OR "breathing difficulties"[All Fields]) OR ("dyspnea"[MeSH Terms] OR "dyspnea"[All Fields] OR ("breathing"[All Fields] AND "difficulty"[All Fields]) OR "breathing difficulty"[All Fields]) OR ("breath"[All Fields] OR "breathe"[All Fields] OR "breathed"[All Fields] OR "breathes"[All Fields] OR "breathings"[All Fields] OR "breaths"[All Fields] OR "respiration"[MeSH Terms] OR "respiration"[All Fields] OR "breathing"[All Fields]) OR "phlegm"[All Fields] OR ("sputum"[MeSH Terms] OR "sputum"[All Fields] OR "sputums"[All Fields]) OR ("cough"[MeSH Terms] OR "cough"[All Fields] OR "coughing"[All Fields] OR "coughs"[All Fields] OR "coughed"[All Fields]) OR ("fibrosi"[All Fields] OR "fibrosing"[All Fields] OR "fibrosis"[MeSH Terms] OR "fibrosis"[All Fields] OR "fibrose"[All Fields] OR "fibroses"[All Fields]) OR ("asthma"[MeSH Terms] OR "asthma"[All Fields] OR "asthmas"[All Fields] OR "asthma s"[All Fields]) OR ("pulmonary disease, chronic obstructive"[MeSH Terms] OR ("pulmonary"[All Fields] AND "disease"[All Fields] AND "chronic"[All Fields] AND "obstructive"[All Fields]) OR "chronic obstructive pulmonary disease"[All Fields] OR ("chronic"[All Fields] AND "obstructive"[All Fields] AND "pulmonary"[All Fields] AND "disease"[All Fields])) OR ("pulmonary disease, chronic obstructive"[MeSH Terms] OR ("pulmonary"[All Fields] AND "disease"[All Fields] AND "chronic"[All Fields] AND "obstructive"[All Fields]) OR "chronic obstructive pulmonary disease"[All Fields] OR "copd"[All Fields]) OR ("lung"[MeSH Terms] OR "lung"[All Fields] OR "lungs"[All Fields] OR "lung s"[All Fields]) OR ("lung"[MeSH Terms] OR "lung"[All Fields] OR "pulmonary"[All Fields]) OR ("eur med j respir"[Journal] OR "respiratory"[All Fields])) OR ((("impair"[All Fields] OR "impaired"[All Fields] OR "impairement"[All Fields] OR "impairements"[All Fields] OR "impairing"[All Fields] OR "impairment"[All Fields] OR "impairments"[All Fields] OR "impairs"[All Fields]) AND ("fasted"[All Fields] OR "fasting"[MeSH Terms] OR "fasting"[All Fields] OR "fastings"[All Fields] OR "fasts"[All Fields]) AND ("glucose"[MeSH Terms] OR "glucose"[All Fields] OR "glucoses"[All Fields] OR "glucose s"[All Fields])) OR ("glucose intolerance"[MeSH Terms] OR ("glucose"[All Fields] AND "intolerance"[All Fields]) OR "glucose intolerance"[All Fields] OR ("impaired"[All Fields] AND "glucose"[All Fields] AND "tolerance"[All Fields]) OR "impaired glucose tolerance"[All Fields]) OR (("impair"[All Fields] OR "impaired"[All Fields] OR "impairement"[All Fields] OR "impairements"[All Fields] OR "impairing"[All Fields] OR "impairment"[All Fields] OR "impairments"[All Fields] OR "impairs"[All Fields]) AND ("glucose"[MeSH Terms] OR "glucose"[All Fields] OR "glucoses"[All Fields] OR "glucose s"[All Fields])) OR (("glucose"[MeSH Terms] OR "glucose"[All Fields] OR "glucoses"[All Fields] OR "glucose s"[All Fields]) AND ("intolerabilities"[All Fields] OR "intolerability"[All Fields] OR "intolerable"[All Fields] OR "intolerably"[All Fields] OR "intolerance"[All Fields] OR "intolerances"[All Fields] OR "intolerant"[All Fields] OR "intolerants"[All Fields])) OR ("glucose intolerance"[MeSH Terms] OR ("glucose"[All Fields] AND "intolerance"[All Fields]) OR "glucose intolerance"[All Fields]) OR "T2D"[All Fields] OR "T2DM"[All Fields] OR ("hyperglycaemia"[All Fields] OR "hyperglycemia"[MeSH Terms] OR "hyperglycemia"[All Fields] OR "hyperglycaemias"[All Fields] OR "hyperglycemias"[All Fields] OR "hyperglycemia s"[All Fields]) OR ("Non"[All Fields] AND ("insulin"[MeSH Terms] OR "insulin"[All Fields] OR "insulin s"[All Fields] OR "insuline"[All Fields] OR "insulinic"[All Fields] OR "insulinization"[All Fields] OR "insulinized"[All Fields] OR "insulins"[MeSH Terms] OR "insulins"[All Fields])) OR "Noninsulin"[All Fields] OR "NIDD"[All Fields] OR ("diabetes mellitus, type 2"[MeSH Terms] OR "type 2 diabetes mellitus"[All Fields] OR "niddm"[All Fields] OR "niddms"[All Fields]) OR ("diabetes mellitus, type 2"[MeSH Terms] OR "type 2 diabetes mellitus"[All Fields]) OR ("type"[All Fields] AND "2"[All Fields]) OR ("diabetes mellitus"[MeSH Terms] OR ("diabetes"[All Fields] AND "mellitus"[All Fields]) OR "diabetes mellitus"[All Fields])) OR ((("decrease"[All Fields] OR "decreased"[All Fields] OR "decreases"[All Fields] OR "decreasing"[All Fields]) AND "gfr"[All Fields]) OR "gfr"[All Fields] OR ("glomerular filtration rate"[MeSH Terms] OR ("glomerular"[All Fields] AND "filtration"[All Fields] AND "rate"[All Fields]) OR "glomerular filtration rate"[All Fields]) OR (("kidney glomerulus"[MeSH Terms] OR ("kidney"[All Fields] AND "glomerulus"[All Fields]) OR "kidney glomerulus"[All Fields] OR "glomerular"[All Fields]) AND ("filtrate"[All Fields] OR "filtrated"[All Fields] OR "filtrates"[All Fields] OR "filtrating"[All Fields] OR "filtration"[MeSH Terms] OR "filtration"[All Fields] OR "filtrations"[All Fields])) OR ("kidney diseases"[MeSH Terms] OR ("kidney"[All Fields] AND "diseases"[All Fields]) OR "kidney diseases"[All Fields]) OR ("polycystic kidney diseases"[MeSH Terms] OR ("polycystic"[All Fields] AND "kidney"[All Fields] AND "diseases"[All Fields]) OR "polycystic kidney diseases"[All Fields] OR ("polycystic"[All Fields] AND "kidney"[All Fields] AND "disease"[All Fields]) OR "polycystic kidney disease"[All Fields]) OR ("kidney diseases"[MeSH Terms] OR ("kidney"[All Fields] AND "diseases"[All Fields]) OR "kidney diseases"[All Fields] OR ("kidney"[All Fields] AND "disease"[All Fields]) OR "kidney disease"[All Fields]) OR ("kidney"[MeSH Terms] OR "kidney"[All Fields] OR "kidneys"[All Fields] OR "kidney s"[All Fields]) OR "CKD"[All Fields] OR ("renal insufficiency, chronic"[MeSH Terms] OR ("renal"[All Fields] AND "insufficiency"[All Fields] AND "chronic"[All Fields]) OR "chronic renal insufficiency"[All Fields] OR ("chronic"[All Fields] AND "kidney"[All Fields] AND "disease"[All Fields]) OR "chronic kidney disease"[All Fields])))) AND ((2020/1:3000/12/12[pdat]) AND (english[Filter]))
